# Costs of Conjunctivitis Outbreak, Réunion Island, France

**DOI:** 10.3201/eid2401.170916

**Published:** 2018-01

**Authors:** Laurent Filleul, Frederic Pagès, Guy-Noel Chan Wan, Elise Brottet, Pascal Vilain

**Affiliations:** Santé Publique France, French National Public Health Agency, Saint-Denis, Réunion, France (L. Filleul, F. Pagès, E. Brottet, P. Vilain);; Agence Régionale de Santé Océan Indien, Regional Public Health Authority, Saint-Denis (G.-N.C. Wan)

**Keywords:** Syndromic surveillance, outbreak, conjunctivitis, French national health insurance, NHI, Oscour network, International Classification of Diseases, SNIIR-AM, South-West Indian Ocean, Réunion Island, France, Madagascar, Mauritius, Cire OI

## Abstract

During January–April 2015, a major outbreak of conjunctivitis on Réunion Island caused a large public health impact. On the basis of general practitioner consultations, emergency department visits, and eye medication sales during the 13-week epidemic, we estimated a total healthcare cost of €3,341,191 from the outbreak.

During January–April 2015, a major outbreak of acute hemorrhagic conjunctivitis occurred on Réunion Island, causing a heavy impact on the national healthcare system of France (*1*). Réunion Island, a French overseas administrated territory, is located in the Indian Ocean between Madagascar and Mauritius; it has a surface area of 2,512 km^2^ and a population of ≈840,000 (1.3% of France’s population, including the nation’s overseas territories; https://www.insee.fr/fr/statistiques/2119468). 

The island is included in the national health insurance (NHI) program of France. Réunion Island’s health system is similar to that of France; however, most patients on the island do not pay provider health fees directly. NHI pays the general practitioner (GP), the pharmacist, or hospital. Rarely, the patients pay for the GP consultations and emergency department (ED) visits, but these costs will be refunded to the patients by the NHI. Healthcare costs are higher (≈30%) on the island than in mainland France. In 2015, total health care expenditures in Réunion Island were €2.561 billion; which is 1.6% of France’s healthcare spending (≈€163 billion) for that year.

A syndromic surveillance system, the Organisation de la surveillance coordonnée des urgences (Organization of coordinated emergency surveillance [OSCOUR]) network, is based on data collected by all EDs across the country, including in French overseas territories (*2*). Data are collected daily directly from patients’ computerized medical files that are completed during medical consultations. For each ED visit, patient age, sex, city of residence, and the diagnosis are recorded. This enables analysis by syndromic groups, age groups, and geographic areas. The diagnosis is categorized according to the International Classification of Diseases, 10th edition (ICD-10; http://www.icd10data.com/). Public health indicators are routinely monitored by using temporal and spatiotemporal analyses, including the number of ED visits for conjunctivitis (ICD-10 code B30 and subcodes, code H10 and subcodes, and code H11 and subcodes).

At the end of January 2015, by using spatiotemporal analysis of data from the OSCOUR network, we detected a cluster of conjunctivitis cases in the western part of the island that occurred during January 26–February 1 (week 5 of 2015). We organized conjunctivitis surveillance within the framework of an existing sentinel project involving 56 volunteer GPs located throughout the island who reported weekly to the Indian Ocean regional institute for public health surveillance agency, known as Cire OI (*3*).

The outbreak on Réunion Island began during week 5 then quickly spread throughout the island and ended in week 17 (end of April) of 2015. Data from ED visits show that all age groups were affected. By using the GP sentinel network and NHI data (*1*), we estimated the total number of GP consultations for conjunctivitis on the island to be 100,094. During this outbreak, we sent regular epidemiologic updates to health professionals to inform them of the ongoing epidemiologic situation and available preventive measures. Health authorities also published a press release for the general public.

On the basis of these data and the major impact for public health, we estimated the cost of this outbreak. We compiled the cost of different indicators: GP consultations, ED visits, and eye medication sales. On Réunion Island, a GP consultation fee of €27.60 and an ED visit fee of €52.60 are reimbursed by NHI. For medicated eye drop sales, we extracted data (number of sales by week and cost) from France’s NHI information system, SNIIR-AM (*4*). During the outbreak period, 187,126 medicated eye drop kits were purchased and reimbursed, at a total cost of €566,443. For activity related to conjunctivitis, the cost for GP consultations was €2,762,597 and for ED visits was €12,151 (Table). During weeks 5–17, the health care cost was estimated at €3,341,191. The total cost is underestimated, however, because it did not include costs to individuals and businesses, including sick leave, work absenteeism of parents for sick children, and some persons who had conjunctivitis but did not consult a physician. 

**Table:** Weekly volume and total costs of medicated eye drop sales, consultations with GPs, and ED visits for conjunctivitis outbreak, Réunion Island, France, January–April, 2015*

Epidemiologic week	No. eye drop sales	No. GP consultations for conjunctivitis	No. ED visits for conjunctivitis	Total cost of eye drop sales, €	Total cost of GP consultations, €	Total cost of ED visits, €
5	7,126	2,641	19	21,206	72,887	999
6	6,818	1,937	9	20,198	53,453	473
7	10,379	3,537	17	31,199	97,617	894
8	14,079	7,439	17	42,646	205,326	894
9	25,831	13,845	33	78,083	382,108	1,736
10	27,345	20,895	41	82,198	576,711	2,157
11	31,866	20,648	21	101,453	569,892	1,105
12	15,339	9,141	15	46,990	252,279	789
13	14,726	6,954	17	43,034	191,921	894
14	11,049	4,832	13	32,717	133,371	684
15	8,180	3,369	17	24,166	92,977	894
16	8,109	2,593	9	24,040	71,574	473
17	6,279	2,264	3	18,514	62,479	158
Total by category	187,126	100,094	231	566,443	2,762,597	12,151
Total costs				3,341,191		

*ED, emergency department; GP, general practitioners.

These data demonstrate that acute outbreaks of illness caused by nonfatal agents can have substantive public health and economic impact. In France, where medical costs are reimbursed by the state, an outbreak of this magnitude, even if virulence is negligible, should be examined thoroughly. Information for the public and health professionals should be strengthened by recurring prevention campaigns with a focus on hygiene, such as washing hands frequently; avoiding rubbing the eyes; covering one’s mouth and nose when coughing or sneezing; and avoiding sharing linen, towels, or any objects owned by affected persons.
